# Comparative genomic hybridizations reveal absence of large *Streptomyces coelicolor *genomic islands in *Streptomyces lividans*

**DOI:** 10.1186/1471-2164-8-229

**Published:** 2007-07-10

**Authors:** Karthik P Jayapal, Wei Lian, Frank Glod, David H Sherman, Wei-Shou Hu

**Affiliations:** 1 Department of Chemical Engineering and Materials Science, University of Minnesota, 421 Washington Ave. SE., Minneapolis, MN 55455, USA; 2 Life Sciences Institute, Departments of Medicinal Chemistry, Chemistry, Microbiology & Immunology, University of Michigan, 210 Washtenaw Ave., Ann Arbor, MI 48109, USA; 3 Abbott Bioresearch Center, 100 Research Drive, Worcester, MA 01605, USA; 4 Fonds National de la Recherche, 6 rue Antoine de Saint-Exupéry, L-1017 Kirchberg, Luxembourg

## Abstract

**Background:**

The genomes of *Streptomyces coelicolor *and *Streptomyces lividans *bear a considerable degree of synteny. While *S. coelicolor *is the model streptomycete for studying antibiotic synthesis and differentiation, *S. lividans *is almost exclusively considered as the preferred host, among actinomycetes, for cloning and expression of exogenous DNA. We used whole genome microarrays as a comparative genomics tool for identifying the subtle differences between these two chromosomes.

**Results:**

We identified five large *S. coelicolor *genomic islands (larger than 25 kb) and 18 smaller islets absent in *S. lividans *chromosome. Many of these regions show anomalous GC bias and codon usage patterns. Six of them are in close vicinity of tRNA genes while nine are flanked with near perfect repeat sequences indicating that these are probable recent evolutionary acquisitions into *S. coelicolor*. Embedded within these segments are at least four DNA methylases and two probable methyl-sensing restriction endonucleases. Comparison with *S. coelicolor *transcriptome and proteome data revealed that some of the missing genes are active during the course of growth and differentiation in *S. coelicolor*. In particular, a pair of methylmalonyl CoA mutase (*mcm*) genes involved in polyketide precursor biosynthesis, an acyl-CoA dehydrogenase implicated in timing of actinorhodin synthesis and *bldB*, a developmentally significant regulator whose mutation causes complete abrogation of antibiotic synthesis belong to this category.

**Conclusion:**

Our findings provide tangible hints for elucidating the genetic basis of important phenotypic differences between these two streptomycetes. Importantly, absence of certain genes in *S. lividans *identified here could potentially explain the relative ease of DNA transformations and the conditional lack of actinorhodin synthesis in *S. lividans*.

## Background

*Streptomyces *spp. include some of the world's most prolific producers of naturally occurring bioactive molecules, many of which are in current therapeutic use [1]. These soil-dwelling filamentous bacteria exhibit a remarkably complex life style. Emerging from uni-genomic spores, they colonize the nutrient layer and eventually surface as multi-genomic aerial hyphae, often synthesizing secondary metabolites in the process. The extraordinary diversity of secondary metabolite gene clusters found in these microbes is likely the result of their existence in hostile ecological niches and consequent genomic evolutionary processes including large scale rearrangements, insertions and deletions to cope with these exigencies. The likelihood of these events are further enhanced by the unusual propensity of *Streptomyces *to undergo spontaneous recombination events especially at chromosome extremities [2,3]. Multiple occurrences of such events over the course of millions of years lead to eventual speciation.

Much of the current knowledge of streptomycetes is based largely on the foundations of genetic and genomic studies conducted in *S. coelicolor *A3(2) [4]. *S. coelicolor *is known for its ability to synthesize pigmented metabolites that serve as excellent phenotypic markers in genetic studies. As a model organism, its genome became the first among streptomycetes to be completely sequenced [5]. Nevertheless, *S. lividans*, a close relative of *S. coelicolor *is almost exclusively considered as the preferred host, among actinomycetes, for heterologous protein expression [6-8]. The primary reasons attributed for this include a significantly relaxed restriction-modification system which enhances exogenous DNA uptake [9] and considerably attenuated endogenous protease activity leading to improved product recovery from *S. lividans *[7]. Notable examples of biologically active heterologous protein productions in *S. lividans *include proteins of eukaryotic origins like human T-cell receptor CD4 [10], tumor necrosis factor-α [11], human interleukin [12] and salmon calcitonin [13] as well as bacterial proteins like mycobacterial antigens with appropriate glycosylation patterns [14]. Leucotropin™ – a recombinant therapeutic agent used in treatment of Hodgkin's disease is commercially produced through *S. lividans *fermentation (Cangene, Winnipeg, Canada).

The 16S rRNA sequences of *S. coelicolor *and *S. lividans *share > 99.5% identity. Historically, the two species have been distinguished by the inability of the latter to produce the deep blue antibiotic, actinorhodin under many conditions. In addition, *S. lividans *(1) fails to methylate its own DNA or restrict exogenous methylated DNA (2) possesses diminished extracellular protease activity (3) lacks a mechanism to degrade agar and (4) forms unstable *φ*C31 lysogens [4].

Despite considerable research and economic interests in *S. lividans *and the availability of complete genome sequence of *S. coelicolor*, there have been surprisingly few systematic studies comparing the genome compositions of the two species. Early work by Leblond *et al*. using pulse-field gel electrophoresis (PFGE) and Southern hybridizations with restriction-fragment linking cosmid probes revealed an essentially similar genomic organization in the two species with identical ordering of the cosmid sequences [15]. More recently, Zhou *et al*. deduced the absence of a ~90 kb *S. lividans *genomic island in *S. coelicolor *through analysis of a DNA modification deficient *S. lividans *derivative ZX7 [16].

In the post-genomic era, DNA microarrays have emerged as the tool of choice for genome scale comparisons of closely related organisms [17,18]. Comparative genomic hybridizations (CGH) using microarrays have already demonstrated their utility in characterization of pathogenicity islands and drug resistance factors in *Yersinia pestis *[19], *Vibrio cholerae *[20], *Staphylococcus aureus *[21] and *Mycobacterium tuberculosis *[22] among many others. Elsewhere, they have been used to assess genome plasticity and microbial evolution [23]. Among *Streptomyces*, microarrays have been previously used to detect gross genomic duplications and presence of long terminal repeats at chromosome ends in different strains of *S. coelicolor *[24]. In this study, we performed a microarray-based whole genome comparison to identify *S. coelicolor *M145 genes absent or divergent in *S. lividans *TK21. Expression levels of these genes are analyzed and relevant observations are discussed in the context of known phenotypic differences that arose during the recent microevolution of these two species.

## Results

### Genome-scale comparison of *S. coelicolor *and *S. lividans*

**Figure 1:**
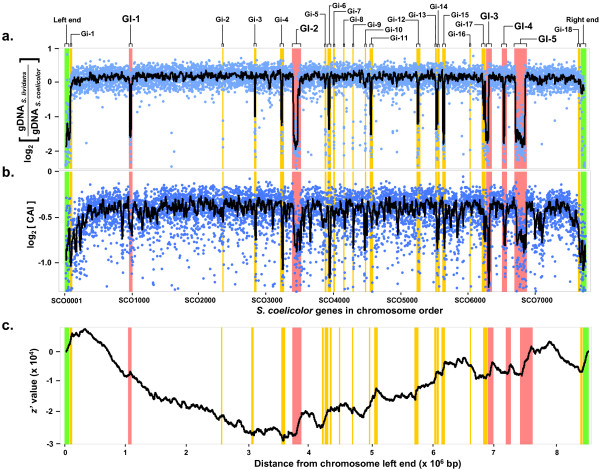
Comparative analysis of *S. coelicolor *M145 – *S. lividans *TK21 chromosomes and genomic features in *S. coelicolor*. **(*a*) **Log_2 _hybridization ratio as a function of *S. coelicolor *ORFs. Low values imply absence or divergence of gene in *S. lividans*. Experimental data (blue circles) and smoothened data using median of a 19-gene sliding window (thick line) are shown. Large genomic islands (red), smaller islets (orange) and *S. coelicolor *specific chromosome end regions (green) identified in this study are marked with filled bars. The sizes of the bars approximate the extent of each island. GI and Gi numbers corresponding to those in Table 1 are indicated. Note that the smoothened curve, shown here for ease of visualization, will not show a dip in hybridization ratios for genomic islets with fewer than 10 genes. **(*b*) **Logarithm of codon adaptation index (CAI) as a function of *S. coelicolor *ORFs. Low log_2 _CAI implies sub-optimal codon usage. **(*c*) ***z*' value plotted as a function of distance from chromosome left end. A sharp increase in *z*' value indicates presence of an AT-rich region. The figure shows that there exists a good correlation between presence of genomic islands, codon usage bias and local GC content.

We had reported earlier the construction of a whole-genome PCR-product based *S. coelicolor *microarray with probes for more than 95% of the predicted ORFs [25]. Comparative genomic hybridizations between *S. coelicolor *M145 and *S. lividans *TK21 using this microarray revealed extensive homology between the two chromosomes. This is apparent from Figure 1a showing the majority of ORFs with relatively similar signal intensity levels from both *S. coelicolor *and *S. lividans *gDNA channels (i.e. log_2 _of hybridization signal ratio close to zero). The observation concurs well with earlier reports of genome-scale conservation between the two species based on restriction fragment based linkage maps [15]. Genes with log_2 _hybridization ratios less than the overall mean (of all genes) minus one standard deviation were designated as potentially absent or divergent in *S. lividans*. Of 7579 *S. coelicolor *genes probed in our array, we found that only ~8% fell in this category. Most often, these genes are clustered in localized regions of the chromosome. The clusters themselves, however, are scattered extensively across the entire length of the chromosome, albeit with a definite bias toward the right half. The absence of these clusters in *S. lividans *were confirmed by PCR with primers flanking the missing region (refer to supplementary material for details). Based on the size of these clusters and gene order in *S. coelicolor *M145, we classified the resident genes as belonging to either one of 5 large genomic islands (GI) (≥25 kb) or 18 smaller islets (Gi) (< 25 kb). These are in addition to the two chromosome ends and about 70 other genes scattered across the chromosome which, despite having low *S. lividans *gDNA signal, did not satisfy the three-adjacent-gene criteria used here to define a genomic island (see Methods section). The chromosome ends were not termed as genomic islands because large-scale DNA rearrangements are known to occur in the terminal regions leading to difficulties in ascertaining the exact boundaries of islands. Also PCR verification using flanking primers was impossible in such cases.

To assess whether these genomic deletions/insertions are consequences of horizontal gene transfer from distant species, we examined each island for characteristic features that define laterally acquired elements. Hallmarks of laterally acquired element include some or all of the following features – (1) unusual GC bias (2) anomalous codon usage (3) presence in the 3' end of tRNA genes (4) tendency to be flanked by direct repeats and (5) inclusion of mobile genetic elements [26,27]. We therefore, sought to evaluate the GC bias and codon usage pattern of genes across the entire genome of *S. coelicolor*. We adopted a windowless cumulative GC plot based on *z' *curve (see Methods section) to evaluate genome-wide GC variation. This approach is more robust, gives higher resolution and has been shown to be useful when the traditional sliding-window based GC plots fail [28]. A sharp rise in the *z' *value at a localized region in the chromosome implies an unusually AT-rich segment. For assessing codon usage patterns, we calculated the Codon Adaptation Index (CAI), a measure of codon optimality [29], for every ORF with respect to the most frequent codons occurring in *S. coelicolor*. CAI values range from 0 to 1.0 with higher values indicating more optimal codon usage. Both the cumulative GC plot and CAI plot revealed excellent correlation with regions identified using microarrays (Figure 1b and 1c). In particular, 18 out of 23 islands/islets identified from microarray analysis have an average CAI ≤ 0.60 – a significant departure from an average of 0.73 for the whole genome. There were, however, some regions with low CAI that were not identified as absent in *S. lividans*. Although, it is quite possible that these segments might have been laterally acquired, our results suggest that those gene transfer events occurred before the evolutionary divergence of these two species. In addition, six of the 23 regions are located at 3' ends of tRNA genes while nine are flanked by near-perfect direct repeat sequences. Some of these repeat sequences had significant homology to the *att *sites of certain plasmids or phage related elements. For example, the repeat sequences flanking Gi-4, Gi-6, Gi-11 and GI-5 resembled the *att *sites of actinophage VWB, plasmid pSG1 (*S. griseus*), pSLP2 (*S. lividans*) and pMB400 (*Bacillus megaterium*) respectively. The list of genomic islands and islets identified and their associated features are shown in Table 1. Taken together, these results suggest that many of these segments are recent evolutionary incorporations into the *S. coelicolor *chromosome.

**Table 1:** Characteristics of genomic islands (GIs) and islets (Gis) identified in this study

**Element **^*a*^	**Size (kb)**	**Number of ORFs**	**% GC Content **^*b*^	**Average CAI (Percent genes with low CAI)**^*c*^	**tRNA in Vicinity**	**Flanking Direct Repeats**	**Mobile Genetic Elements**
Chromosome left end; SCO0001-0081	69.6	81	69.5	0.58 (59%)	-	-	8 transposon related genes
Gi-1; SCO0090-0099	6.6	10	67.1	0.56 (67%)	-	-	6 transposon related genes
**GI-1; SCO0979-1000**	25.9	22	70.7	0.67 (23%)	-	-	1 phage related integrase
Gi-2; SCO2381-2384	6.4	4	68.4	0.57 (75%)	-	-	-
Gi-3; SCO2862-2871	8.6	10	65.9	0.54 (78%)	-	-	1 gene similar to phage replication regulator
Gi-4; SCO3250-3270	14.0	21	66.2	0.54 (80%)	Arg-tRNA CCT	43 bp perfect repeat	Mostly plasmid (pSAM2) related genes
**GI-2; SCO3437-3506**	75.0	70	68.7	0.60 (55%)	-	61 bp repeat (6 mismatches)	5 transposon related genes
Gi-5; SCO3929-3937	9.2	8	68.0	0.59 (50%)	-	-	pSAM2 integration site, some plasmid functions
Gi-6; SCO3980-3998	10.7	19	65.8	0.44 (100%)	Ser-tRNA TGA	106 bp repeat (8 mismatches)	pSAM2 insertion element
Gi-7; SCO4060-4066	5.2	7	66.1	0.43 (100%)	Ser-tRNA GGA	88 bp repeat (4 mismatches)	2 transposon related genes
Gi-8; SCO4210-4213	3.8	4	66.8	0.50 (75%)	-	19 bp perfect repeat	Some phage related genes
Gi-9; SCO4346-4350	7.0	5	66.9	0.55 (75%)	-	-	-
Gi-10; SCO4533-4537	4.3	5	72.1	0.70 (0%)	-	-	-
Gi-11; SCO4615-4631	15.9	17	68.3	0.62 (31%)	Tyr-tRNA GTA	112 bp repeat (1 mismatch)	Possible SLP1 insertion
Gi-12; SCO5327-5350	21.3	24	65.3	0.67 (55%)	Arg-tRNA CCG	114 bp repeat (4 mismatches)	pSAM2 integration, plasmid/phage related genes
Gi-13; SCO5605-5620	12.9	16	67.0	0.58 (67%)	-	-	All phage related genes
Gi-14; SCO5632-5644	10.4	13	63.8	0.44 (100%)	-	52 bp repeat (5 mismatches)	Some plasmid related functions
Gi-15; SCO5718-5735	21.8	18	68.1	0.56 (72%)	-	-	-
Gi-16; SCO6120-6124	2.9	5	70.0	0.67 (25%)	-	-	-
Gi-17; SCO6314-6338	23.6	23	70.2	0.59 (70%)	-	-	2 transposon related genes
**GI-3; SCO6353-6405**	56.6	53	68.6	0.56 (61%)	-	-	4 transposon related genes
**GI-4; SCO6625-6642**	30.4	18	68.6	0.56 (65%)	-	-	Few phage related genes
**GI-5; SCO6806-6953**	153.3	148	69.0	0.60 (53%)	Pro-tRNA GGG	44 bp perfect repeat	1 transposon related gene at end
Gi-18; SCO7795-7802	4.2	8	65.2	0.48 (86%)	-	-	3 transposon related genes
Chromosome right end; SCO7827-7845	20.4	19	70.1	0.55 (83%)	-	-	1 transposon related gene;

^*a *^Large genomic islands (≥25 kb) are in bold face

^*b *^Percentage GC content within each genomic island; compare with 72.1% average GC content of whole genome

^*c *^Compare the average CAI of ORFs within each segment with 0.73, the average CAI of all genes in *S. coelicolor*. Percent genes with low CAI is the fraction of genes in each island with CAI values lower than global mean – standard deviation (= 0.625)

### Analysis of GIs and resident genes

A total of 625 genes reside within the *S. coelicolor *genomic islands/islets identified above. They were categorized based on their functional assignments (Table 2). Unsurprisingly, more that two-thirds of laterally acquired elements in *S. coelicolor *are absent in *S. lividans*. Mobile genetic elements like integrative plasmids, bacteriophages and transposon related genes constitute this class. Other genes missing in *S. lividans *are comprised largely of hypothetical and periplasmic/exported proteins although a significant fraction belongs to a diverse set of functional categories shown in Table 2.

**Table 2:** Functional classification of *S. coelicolor *M145 genes missing or divergent in *S. lividans *TK21

**Function**^*a*^	**Total Present**^*b*^	**Number Missing/Divergent**	**Percent Missing/Divergent**
Cell processes	800	32	4 %
Macromolecule metabolism	496	20	4 %
Amino acids biosynthesis	123	3	2 %
Nucleotide biosynthesis	30	0	0 %
Ribosomal constituents	67	0	0 %
Biosynthesis of cofactors and carriers	118	0	0 %
Central intermediary metabolisms	111	4	4 %
Degradation of small molecules	200	6	3 %
Energy metabolism	189	1	1 %
Fatty acid and phosphatidic acid biosynthesis	56	4	7 %
Secondary metabolism	277	3	1 %
Periplasmic, exported or lipoproteins	1318	84	6 %
Two-component systems	165	7	4 %
RNA polymerase core enzyme binding	88	3	3 %
Regulatory proteins	673	39	6 %
Protein kinases	39	1	3 %
Laterally acquired elements	139	95	68 %
Not classified	565	30	5 %
Hypothetical proteins	2371	293	12 %
			
**Total**	**7825**	**625**	**8%**

^*a *^protein classification scheme derived from EcoCyc database; downloaded from

^*b *^Total number of proteins present in *S. coelicolor *chromosome in each category

GI-1 (22 ORFs; ~26 kb) consists of a variety of genes including those coding for acetate uptake, amino acid biosynthesis and possible iron uptake systems. Superoxide dismutase – SCO0999 (*sodF2*), an Fe-dependent antioxidant was previously hypothesized to have been acquired by horizontal gene transfer [30]. SCO0981-83 constitutes an acetate uptake system with a DNA-binding regulator (*aceR*), isocitrate lyase (*aceA*) and malate synthase (*aceB2*) genes. Isocitrate lyase and malate synthase are required for a functional glyoxylate pathway which promotes acetate utilization; yet, expression of these genes in *S. cinnamonensis *did not restore growth of acetate-uptake-deficient mutants on acetate as sole carbon source giving rise to the possibility that some, as yet unknown, factors are missing [31]. SCO0997 and SCO0998 are annotated as iron-uptake system proteins – *ftrD *and *ftrE *respectively. However, the presence of an upstream gene (SCO0996) homologous to a lipoprotein found in daptomycin biosynthesis cluster has raised questions about their involvement in iron uptake [32]. This is further corroborated by the presence of chromosomally linked genes homologous to SCO0991/92 (a hypothetical protein and a putative cysteine synthase) near the daptomycin cluster of *S. roseosporus *[33]. If true, the presence of these antibiotic biosynthesis linked genes in a genomic island of *S. coelicolor *would be intriguing in view of evidence for horizontal transfer of antibiotic clusters among streptomycetes isolated from soil [34].

GI-2 (70 ORFs; ~75 kb) includes ORFs encoding a number of transposases and insertion elements. Interspersed within this region are a putative extracytoplasmic function sigma factor (SCO3450), a methyltransferase (SCO3452), a set of putative ABC-type transporters related to spermidine/putrescine transport family (Pot) (SCO3453-56) and an assortment of consecutive genes with various putative metabolic activities (SCO3473-3506). It is noteworthy here that the only extracellular agarase (*dagA*, SCO3471) of *S. coelicolor *maps to this genomic segment. This explains the lack of agarase activity in cultures of *S. lividans *noted elsewhere [35].

GI-3 (53 ORFs; ~57 kb) contains elements of three putative two-component systems (SCO6353/54, SCO6362/63/64 and an orphan kinase SCO6369). The region also encompasses ORFs with bacitracin transport permease domains (SCO6356, 6360, 6378). The last 16 ORFs in this island comprise mainly of mobile genetic elements like transposases and recombinases.

GI-4 (18 ORFs; ~30 kb) contains a phage growth limitation (Pgl) system. This is a defense mechanism against *φ*C31 bacteriophage infections in which an infecting phage undergoes a single burst of attack and the resulting progeny are severely attenuated in subsequent infectious cycles. Two loci, *pglWX *and *pglYZ *– both of which map to this genomic locus – are necessary for the Pgl^+ ^phenotype. Absence of these genes in *S. lividans *leads to unstable *φ*C31 lysogens while complementing them with these elements give rise to Pgl^+ ^colonies [36].

GI-5 (148 ORFs; ~153 kb) is the largest genomic island identified in this study. The size of this cluster implies that it hosts a multitude of genes with varied functions. Importantly, this cluster hosts genes that are involved in various modes of microbial defense. SCO6826/27 encodes a pair of type I modular polyketide synthase genes. In close proximity are two genes (SCO6832/33) coding for subunits of methlymalonyl-CoA mutase – an enzyme that catalyzes conversion of succinyl-CoA to methylmalonyl-CoA, which serves as building blocks for certain polyketide antibiotic synthesis [37]. Two other genes, SCO6929/30 are similar to those involved in lantibiotic biosynthesis. In addition, SCO6835/36/37 belong to the arsenic resistance family. SCO6809/10 are similar to several multi-drug efflux transporters. Also, a probable acyl-CoA dehydrogenase (SCO6938) belonging to this island has been implicated in control of actinorhodin production and timing of sporulation [38]. This observation is particularly interesting considering the extremely low-levels of actinorhodin production [39] and absence (or silencing) of the *ram*-independent developmental pathway in *S. lividans *[40]. Furthermore, two putative DNA methylases (SCO6844 and SCO6885) also map to this locus.

The smaller genomic islets (Gi-1 to Gi-18) also contain several potentially interesting genes. Foremost among them is Gi-15. This islet includes the *bldB *locus (SCO5723) which has been attributed to pleiotropically regulate both antibiotic synthesis and morphological differentiation [41]. Two other genes found in this islet (SCO5722 and SCO5731) encode putative secreted serine proteases. This islet is also unusual in being flanked on one side by a highly expressed *rpsO *gene rather than a tRNA. Among other smaller islets, Gi-12 contains an adenine-specific DNA methylase (SCO5331, *pglS*). This gene has been confirmed to interfere with the Pgl system of GI-4 and might extend resistance to phages other than *φ*C31 [42]. Gi-10 and Gi-11 each encode a methyl specific restriction endonuclease – SCO4213 (*mrr*-like) and SCO4631 (*mcrA*-like) respectively. The rest of the genes in the genomic islands/islets are largely comprised of hypothetical proteins and other genes whose functions are yet to be elucidated.

### Comparison with transcriptome and proteome data

**Figure 2:**
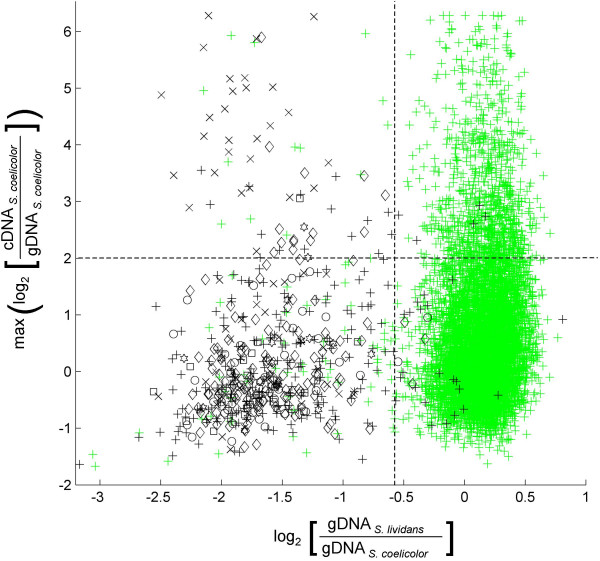
Comparison of CGH and transcriptome data. Plot of log_2 _[gDNA_*Sliv*_/gDNA_*Scoe*_] (x-axis) *vs *max(log_2 _[mRNA_*Scoe*_/gDNA_*Scoe*_]) (y-axis). The y-axis values correspond to the maximum gene expression levels observed considering all analyzed samples (includes both R5^- ^and SMM). Genes belonging to GI-1 (), GI-2 (×), GI-3 (○), GI-4 (□), GI-5 (◇) and all other smaller islets (black +) are shown alongside genes conserved in both *S. coelicolor *and *S. lividans *(green +). Genes in the region left of and above the dotted lines are those that are highly expressed in *S. coelicolor *but absent in *S. lividans*. High expression in this case was defined as max(log_2 _[mRNA_*Scoe*_/gDNA_*Scoe*_]) ≥ 2.0 which corresponds to the top 14 percentile of gene expression values. The plot shows that a large fraction of genes absent in *S. lividans *have low expression levels in *S. coelicolor*. Nevertheless, there are some prominent examples of highly expressed genes within certain *S. coelicolor *genomic islands. This includes several genes from GI-2.

Assuming that these genomic islands have evolved under selective pressure, we asked which, if any, of these genes are actively expressed under normal (laboratory) culture conditions. We chose to analyze mRNA and protein expression data derived from two distinct liquid media – one, an R5-based rich medium with yeast extract and the other, a minimal medium supplemented with casaminoacids (SMM). Due to significant differences in their composition, we expected that they will elicit markedly distinctive gene expression patterns. Data for the R5-based cultures were generated using our microarray and LC-MS experiments (Jayapal *et al*., in preparation) while those for SMM cultures were downloaded from a public repository  or from previously published 2-D gel proteomic analyses [43,44]. Since the microarray hybridizations were performed with *S. coelicolor *gDNA reference, the resulting log_2 _expression ratios (= log_2 _[mRNA_*Scoe*_/gDNA_*Scoe*_]) are an indication of the expression level for each gene [25]. The highest log_2 _expression ratio observed for a given gene over the range of growth phases and conditions analyzed can be taken as its potential for transcription under laboratory conditions. Figure 2 shows this transcriptome expression data plotted against comparative genomics hybridization data. Genes in the top-left region of the plot (marked in Figure 2) are, in general, designated as absent in *S. lividans*, yet highly expressed in *S. coelicolor *at least in some conditions. Note that this region also includes a few genes with quite low log_2 _[gDNA_*Sliv*_/gDNA_*Scoe*_] value but were not designated as absent/divergent in *S. lividans *simply because they did not co-localize as three- or more-gene clusters and hence did not satisfy out absence criteria (see Methods section). Reciprocally, some genes with high log_2 _[gDNA_*Sliv*_/gDNA_*Scoe*_] were assigned to certain genomic islands because of overwhelming evidence of absence from neighboring genes. Many such genes with considerable homology are transposon/plasmid related elements and are probably present elsewhere in the *S. lividans *chromosome. 42 genes from various genomic islands exceeded a threshold of max(log_2 _[mRNA_*Scoe*_/gDNA_*Scoe*_]) value greater than 2.0 indicating appreciably high mRNA expression. 20 of these genes also had an average(log_2 _[mRNA_*Scoe*_/gDNA_*Scoe*_]) > 0 indicating probable constitutive basal expression (data not shown in Figure). Currently, more than half of those 42 genes (23/42) do not have a reasonably descriptive functional annotation.

The most striking observation from the transcriptome analysis was the high expression of ~30 contiguous genes (SCO3473-3506) within GI-2 exclusively in SMM cultures. These genes encode a variety of probable carbohydrate metabolism enzymes including putative aldolase, isomerase, dehydrogenase, beta-galactosidase, sugar kinase and sugar permease; the precise context of these functions remain to be elucidated. Nevertheless, such coordinated but conditional transcription of genes within genomic islands leads one to speculate that, in addition to collective horizontal transfer as an island, they are also regulated in an independent and rather concerted fashion in response to varying environmental stimuli. In addition, the extracellular agarase precursor (SCO3471) in GI-2 is also expressed highly only in SMM cultures.

Several genes in GI-1 are also highly expressed. The superoxide dismutase (SCO0999, *sodF2*) has an average(log_2 _[mRNA_*Scoe*_/gDNA_*Scoe*_]) of over 2.0 in cultures of R5-based medium and was also identified in LC-MS proteomic studies with multiple peptide hits (Jayapal *et al*., in preparation). However, this gene is not as highly expressed in SMM cultures. On the contrary, adjacent genes SCO0985/0993/0995 were identified in the 2-D gel experiments conducted on minimal medium derived samples [43]. Genes in *bldB *locus (Gi-15) are also expressed at moderate to high levels from microarray data (in both rich and minimal medium). This is corroborated by the identification of some of these proteins (SCO5723/24 and SCO5729) in proteomic experiments. Interestingly, SCO5724/25 possibly codes for a novel secretion system [45]. Such transporters are widely associated with pathogenicity islands in many bacteria [26]. Furthermore, phage resistance proteins of the Pgl system (SCO6627/28/37/38; GI-4) were identified in several proteomic experiments. Notably, one of them – SCO6627 (PglX) was identified in multiple forms in the membrane fraction of mycelium undergoing programmed cell death [44]. Likewise, polyketide precursor synthesis enzymes (SCO6832/33, methylmalonyl-CoA mutase subunits in GI-5) were identified in LC-MS experiments of R5 medium samples (Jayapal *et al*., in preparation).

## Discussion

### *S. coelicolor *contains large laterally acquired genomic segments

Our results indicate that over 90% of *S. coelicolor *genes are conserved remarkably well in *S. lividans*. This observation is not surprising considering that even bacteria from other genera, namely *Mycobacterium *and *Thermofibida*, share a significant homology with genes in *Streptomyces *[46]. Punctuating this pattern of widespread conservation are 23 genomic islands comprising > 600 genes that are conspicuously absent in *S. lividans*. Many of these islands exhibit hallmarks of horizontal gene transfer. Although it is generally believed that chromosome extremities are more prone to massive gene flux compared to the central "core" [2], we did not observe a higher occurrence of genomic islands near the chromosome ends (10 genomic islands in the "core" compared to eight in the chromosome "arms"). This could partly be explained for certain islands containing phage-related elements or integrative plasmids that target specific sequences rather than rely on genome instability for chromosome integration. There is at least one report of DNA amplifications in the "core" region of *S. coelicolor *indicating the chromosomal instabilities are not strictly limited to the extremities [47]. Many of these regions (eg. GI-1, GI-4, GI-5, Gi-15) harbor elements that could potentially benefit the host, asserting that they truly are "fitness islands". Such elements include antibiotic biosynthesis genes, drug resistance determinants, phage defense systems, and numerous metabolic functions.

It is notable that 14 of the 23 islands detected in this study were reported as putative laterally acquired regions through *in silico *analysis during sequencing of the *S. coelicolor *genome [5]. Half of those (7/14) were reported precisely as described here; for the rest, the exact boundaries of the islands do not match our results. This does not, however, imply that our results contradict previous observations. One likely possibility is that genes in any extended genomic island predicted by Bentley *et al*., although recently acquired, are well-conserved in *S. lividans *(probably acquired before speciation). Another possibility is that the apparent discrepancies are merely consequences of our strict criteria for absence that eliminated certain marginal cases. On the other hand, all boundary extensions (and new islands) reported in this work provide conclusive experimental evidence of recent gene transfer in those regions.

### Hints for genetic basis of suppressed antibiotic synthesis in *S. lividans*

Over the years, identification of genetic determinants that suppress actinorhodin production in *S. lividans *has aroused considerable interest among researchers. Although numerous mutational and overexpression studies have led to activation of antibiotic synthesis in *S. lividans *[48-52], to our knowledge, none have succeeded in identifying the evolutionary transformation that led to modulation of actinorhodin synthesis. Our results provide two important clues in this regard. One is the absence of a probable acyl-CoA dehydrogenase (SCO6938) in *S. lividans*, whose mutation in *S. coelicolor *caused a marked delay in onset of actinorhodin synthesis as well as aerial mycelium formation [38]. Acyl-CoA dehydrogenases are enzymes associated with breakdown of long-chain fatty acids and might potentially generate precursors for polyketide biosynthesis [53]. Of particular note is a mutant of an acyl-CoA synthetase (another enzyme involved in fatty acid catabolism) in *S. coelicolor *that was severely impaired in actinorhodin synthesis particularly in glucose containing medium [54] – an observation curiously reminiscent of the conditional suppression of actinorhodin biosynthesis in *S. lividans *in glucose containing medium. The other clue comes from the apparent absence of *bldB *(SCO5723) locus in *S. lividans*. At least two other paralogs of *bldB *(SCO3424 and SCO4542) are also known to exist in *S. coelicolor *chromosome, both of which are conserved in *S. lividans*. However, these paralogs do not substitute for *bldB *– *S. coelicolor *mutants of *bldB *fail to erect aerial hyphae and lack actinorhodin production; yet, *S. lividans *appears to be quite capable of forming aerial mycelia and spores. Despite the apparent absence of SCO5723 locus in *S. lividans*, we also noted that a *S. lividans *gene annotated as *bldB *(GenBank accession AF071232) has been cloned and expressed in *E. coli *[55]. A closer look revealed that this *S. lividans bldB *shares only ~82% homology with SCO5723 and hence gave a weak hybridization signal – an observation further corroborated by the failure of stringent Southern hybridizations to reveal the presence of *bldB *in *S. lividans *[56]. Perhaps, this level of similarity is sufficient for most of the functional activity of BldB. Notably, all the conserved residues reported by Eccleston *et al*. [57] are present in both versions of the protein. Nonetheless, our data suggests that this ortholog in *S. lividans *is present at an alternate locus since an entire 17-gene *S. coelicolor *segment (Gi-15) including SCO5723 is absent in *S. lividans*. We note that the other *bldB *paralogs of *S. coelicolor *have a much lesser degree of homology with *S. lividans bldB*.

### Absence of certain endonucleases and proteases in *S. lividans*

Another question of particular interest in this comparative study is what genetic factors make *S. lividans *a preferred host for heterologous protein expression? As mentioned earlier, the two contributing factors in this regard are the absence of a strong restriction-modification system and diminished extracellular protease activity. The phage growth limitation (Pgl) system found in GI-4 contains elements required for DNA restriction. Although this explains the unstable *φ*C31 lysogens of *S. lividans*, introduction of this system into *S. lividans *did not impair its ability to uptake methylated DNA [36]. Two other potential candidates – SCO4213 and SCO4631 (both annotated as hypothetical proteins) were identified in a scan for methyl-sensing restriction endonucleases. SCO4213 contains a signature Mrr_cat type II restriction enzyme domain while SCO4631 is similar to an *E. coli *methylcytosine-specific restriction enzyme with an HNH endonuclease domain. In addition, SCO2863 – a putative helicase, also contains an *hsdR*-like type I restriction endonuclease domain. Moreover, the absence of two DNA-methylases, SCO6844 and SCO6885 might explain why DNA obtained from *S. lividans *is readily transformable into other streptomycetes. The attenuated extracellular protease activity in *S. lividans *is, perhaps, explained by the absence of two secreted serine proteases – SCO5722 and SCO5731.

### TTA codon modulated gene expression is a recent evolutionary incorporation

An interesting observation that we noted in our analysis is the unusually high frequency of the rare TTA codon containing genes designated as absent in *S. lividans*. About 30% (43/145) of such genes are absent in *S. lividans *– statistically a much higher frequency compared to only 8% of all genes absent. Considering that AT-rich segments frequently occur in bacterial phages and plasmids [58] and also the fact that occurrences of TTA codons are quite rare in a GC-rich organism like *S. coelicolor*, it is likely that many of these genes are of foreign origin. In fact, a recent report by Chater and Chandra postulated that over 80% of TTA-containing *S. coelicolor *genes were acquired through horizontal gene transfer [46]. They speculated that *bldA *(the sole tRNA that can efficiently translate TTA) might itself have been laterally acquired, and that TTA-modulated protein expression is a very recent evolutionary adaptation. Our experimental results are consistent with their hypothesis.

## Conclusion

Despite the presence of over 600 genes in these *S. coelicolor *genomic islands, we found that over 93% were not highly expressed under typical laboratory conditions analyzed in this study. This is probably due to the fact that these elements require specific environmental stimuli for activation. Experimental conditions covering a wider range of physiological conditions will need to be tested for this purpose. Another rather likely possibility is that our genomic tools were simply not sensitive enough to detect certain physiologically relevant gene expression levels. We also note that genomic islands present in *S. lividans *and absent in *S. coelicolor *could not be identified in this study. Notwithstanding these limitations, our work sheds light into possible genetic determinants contributing to phenotypic differences between *S. coelicolor *and *S. lividans*. More importantly, it lays a strong foundation for identification of specific gene targets in the genetically well-characterized *S. coelicolor *to engineer it for industrial protein or secondary metabolite production processes.

## Methods

### Strains and culture conditions

Spores for *S. coelicolor *M145 and *S. lividans *TK21 were generated on Mannitol-Soy flour or R5 agar [4]. Cultures for genomic DNA preparation were performed in YEME medium with 0.5% glycine supplement at 30°C until early stationary phase.

### Genomic DNA extraction and labeling

Genomic DNA (gDNA) extraction was carried out using Kirby mix procedure as described elsewhere [4]. About 500 μl of 20 μg/μl gDNA was sonicated briefly for 30–40 sec for shearing them to ~500 bp average size (confirmed by gel electrophoresis). The DNA was then labeled with *Label *IT^® ^Cy3 or Cy5 Labeling Kit (Mirus Bio Corp., Madison, WI) according to suppliers instructions.

### Microarray hybridizations and image analysis

Samples containing ~500 ng each of Cy3 and Cy5 labeled gDNA from *S. coelicolor *M145 and *S. lividans *TK21, respectively were hybridized to a whole-genome *S. coelicolor *microarray as described previously [25]. Hybridizations were carried out in triplicate for ~16 hr at 50°C; arrays were washed and scanned using ScanArray5000 (Perkin Elmer, Wellesley, MA). Images were analyzed using GenePix (Axon Instruments, Union City, CA) to obtain raw intensity data for each spot. The median fluorescence intensity from each spot was used for all subsequent analysis.

### Array normalization and data analysis

Raw relative intensity values for each spot were first normalized by scaling one of the gDNA channel signal intensities by a normalization factor to set the total intensity from both channels as equal. Log_2 _hybridization signal ratios were then calculated from normalized intensities as log_2 _[gDNA_*Sliv*_/gDNA_*Scoe*_] and values were averaged using the median from triplicate experiments. Presence of at least three consecutive (in chromosome order) log_2 _[gDNA_*Sliv*_/gDNA_*Scoe*_] values less than its global mean minus one standard deviation was taken as evidence for absence (or divergence) of a contiguous genomic segment in *S. lividans*. Certain marginal cases were thereafter manually reassigned as present/absent in *S. lividans *based on overwhelming trends of neighboring genes. For visualization purposes, median intensity ratios from successive 19-gene sliding windows were plotted as a function of genes in chromosome order.

For transcriptome analysis, cDNA-gDNA based microarray data was normalized using quantile normalization [25].

All microarray data discussed here are available at Gene Expression Omnibus (GEO): Accession – GSE7167 (comparative genomics hybridizations) and GSE7172 (transcriptome data).

### Sequence analysis

Regional GC variations in *S. coelicolor *genome were calculated using *z*' curve method proposed by Zhang and Zhang [59]. Briefly, *z*_*n *_was calculated at genomic location *n *as:

*z*_*n *_= (A_*n *_+ T_*n*_) - (G_*n *_+ C_*n*_), *n *= 0, 1, 2...N

where A_*n*_, T_*n*_, G_*n *_and C_*n *_are the cumulative number of bases A, T, G and C occurring in a sequence from 1^st ^to *n*^th ^base. To amplify the deviations of *z*_*n *_from its average trend, a linear least square fit: *z*_*n *_= *kn *is performed and *z*_*n*_' is calculated as

*z*_*n*_' = *z*_*n *_- *k*_*n*_

Codon Adaptation Index (CAI), a measure of utilization of "optimal codons" was calculated as described previously [29]. First, a relative adaptiveness factor for every codon *i *coding for amino acid *j *is estimated as:

wi,j=fi,j(G)fmax⁡,j(G), for all codons i and amino acids j
 MathType@MTEF@5@5@+=feaafiart1ev1aaatCvAUfKttLearuWrP9MDH5MBPbIqV92AaeXatLxBI9gBaebbnrfifHhDYfgasaacH8akY=wiFfYdH8Gipec8Eeeu0xXdbba9frFj0=OqFfea0dXdd9vqai=hGuQ8kuc9pgc9s8qqaq=dirpe0xb9q8qiLsFr0=vr0=vr0dc8meaabaqaciaacaGaaeqabaqabeGadaaakeaacqWG3bWDdaWgaaWcbaGaemyAaKMaeiilaWIaemOAaOgabeaakiabg2da9maalaaabaGaemOzay2aaSbaaSqaaiabdMgaPjabcYcaSiabdQgaQbqabaGccqGGOaakcqWGhbWrcqGGPaqkaeaacqWGMbGzdaWgaaWcbaGagiyBa0MaeiyyaeMaeiiEaGNaeiilaWIaemOAaOgabeaakiabcIcaOiabdEeahjabcMcaPaaacqGGSaalcqqGGaaicqqGMbGzcqqGVbWBcqqGYbGCcqqGGaaicqqGHbqycqqGSbaBcqqGSbaBcqqGGaaicqqGJbWycqqGVbWBcqqGKbazcqqGVbWBcqqGUbGBcqqGZbWCcqqGGaaicqWGPbqAcqqGGaaicqqGHbqycqqGUbGBcqqGKbazcqqGGaaicqqGHbqycqqGTbqBcqqGPbqAcqqGUbGBcqqGVbWBcqqGGaaicqqGHbqycqqGJbWycqqGPbqAcqqGKbazcqqGZbWCcqqGGaaicqWGQbGAaaa@714B@

where *f*_*i*,*j*_(*G*) is the frequency of occurrence of codon *i *among the set for amino acid *j *across the entire genome *G *and *f*_max,*j*_(*G*) is the corresponding value for the most frequently used codon for amino acid *j*. The CAI for a gene *g *is then calculated as

CAIg=∏i=1Nwi1/N
 MathType@MTEF@5@5@+=feaafiart1ev1aaatCvAUfKttLearuWrP9MDH5MBPbIqV92AaeXatLxBI9gBaebbnrfifHhDYfgasaacH8akY=wiFfYdH8Gipec8Eeeu0xXdbba9frFj0=OqFfea0dXdd9vqai=hGuQ8kuc9pgc9s8qqaq=dirpe0xb9q8qiLsFr0=vr0=vr0dc8meaabaqaciaacaGaaeqabaqabeGadaaakeaaieaacqWFdbWqcqWFbbqqcqWFjbqsdaWgaaWcbaGaem4zaCgabeaakiabg2da9maarahabaGaem4DaC3aa0baaSqaaiabdMgaPbqaaiabigdaXiabc+caViabd6eaobaaaeaacqWGPbqAcqGH9aqpcqaIXaqmaeaacqWGobGta0Gaey4dIunaaaa@3F07@

where *N *is the number of amino acids in gene *g*. The algorithms for both cumulative GC plot and CAI plot were implemented using Matlab 7.0 with bioinformatics toolbox.

### PCR verification of islands absent in *S. lividans*

The absence of genomic islands in *S. lividans *were verified by PCR using primers flanking each island. Primers were chosen so as to fall in or outside of the DNA probe segment used in microarray to improve chances of amplification. The PCRs were conducted using GC-rich™ PCR system or Expand™ long template PCR system (Roche Applied Science, Indianapolis, IN) with 2% DMSO. Successful amplification of a relatively small product from *S. lividans *indicates absence of the intervening genomic island in each case.

## Authors' contributions

KPJ analyzed and interpreted comparative genomic hybridization data, performed sequence analysis, carried out PCR verifications, performed transcriptomic and proteomic experiments and drafted the manuscript. WL participated in preliminary data analysis including data normalization and interpretations. FG conceived of the study and carried out comparative genomic hybridizations. DHS and WSH participated in discussions, critical review of results presented here and assisted in manuscript preparation. All authors have read and approved the final manuscript.

## Supplementary Material

Additional file 1 PCR verification for genomic islands – primers used and gel images. Details of primers chosen for PCRs to verify the absence of genomic islands in *S. lividans *and gel electrophoresis images of PCR products in each case. Click here for file

Additional file 2 Table of microarray data and GI designations of genes probed. Contains comparative genomic microarray hybridization ratios from three replicate experiments, designations of presence/absence in *S. lividans*, GI designations, CAI values and maximum/average expression in transcriptome data for each gene. Click here for file
