# Supplementary material for: Comparative genomic hybridizations reveal absence of large Streptomyces coelicolor genomic islands in Streptomyces lividans
Source: BMC Genomics. 2007 Jul 10;8:229. doi: 10.1186/1471-2164-8-229 (PMC1934918; doi:10.1186/1471-2164-8-229)
Supplement: Additional file 1 — PCR verification for genomic islands – primers used and gel images. Details of primers chosen for PCRs to verify the absence of genomic islands in S. lividans and gel electrophoresis images of PCR products in each case. [file 1471-2164-8-229-S1.pdf]

PCR VERIFICATION FOR GENOMIC ISLANDS – PRIMERS USED AND GEL IMAGES

Table S1: Primers used for PCR verification of Genomic Islands

Genomic island[a]	Left primer (5'-3')	Right primer (5'-3')
**GI-1**	TAGCTGATGAGGATCACCAGGTCAC	AAGATCGCGAGTATCGAGACCTATC
Gi-2	TGCTCGGGTACGTGTTCAG	AGGTGCTGGTGTGGGAGAT
Gi-3	GGTGAAGTAGATGTGGTTCATCAGG	ACCCGTACCAGATGGTCAAGGAC
Gi-4	GCTGTAGTCGAGGACGCTCAGGT	GGTGGAGTACGACCAGGAGTATGTC
**GI-2**	GCCGGGAGACCGACCTCTAC	CGGTGCCGGTCTTTCGAC
Gi-5	ATCACAGAGCAGTTCGGTGGTACG	GCTGGTTGTAGAAGACGAAGTCACC
Gi-6	GAGGTTCGTGATCGACGCCTACT	GAGAAGGTGATGGTGTACGTGAAGG
Gi-7	ATGAACGAGTCGGCACATTCCAC	CTGAACAGGTACGCGTGATTGAC
Gi-8	AACTCAACGCCGAGTTCAAG	GGTGGTCTCCTTCGAGATGT
Gi-9	AGAGTGGGCCAGCAGAACT	GCGTAGTCGAGTGCTGACC
Gi-10	ACCTACTCCCCGGTCTTCAC	ACGAACTCACCAAGCAGGTC
Gi-11	ACCTTCGAGACGATGTCGAAACTG	GACGAGATCTTCGACGTCTACCTG
Gi-12	TAGGCTCACTCGAGCTATCCATCCT	ACTTCCAGACACTCGGCACCATC
Gi-13	ATCCAGACCCACGGAGGAG	GTCTTGTACGACCGCCACAG
Gi-14	CTCGAAGTAGGACGTGGAGAACAG	GAATGTGAGTTCTCCGGGCTTAG
Gi-15	CTGGGCGTAGGGCTGGTTG	ACCATGGTGTCGTCGTCCAGGTA
Gi-16	ATCAGCCCGCAGGAGTACC	CGGTGACGTAGACCAGTTCG
Gi-17	CGGTCGCCTGGACCTCAAC	GGCTGACCCTCGCCTACCTG
**GI-3**	ATGCGCGTGGTGTTCGTG	GCGTTCCAGCCCGAGTCC
**GI-4**	AAGGAGAAGTGGTCCCTGAAAGC	AGGCTGAACTCCCAGGTCCTGTC
**GI-5**	CTCGAACACTCCTCGGACGACTT	GAAGTAGGAGGCGCAGTAGACCAG

[a] PCR products for Gi-1 and Gi-18 which are close to the chromosome ends could not be amplified (possibly due to genome rearrangements that might have occurred in *S. lividans*). Hence those primers are not shown here.

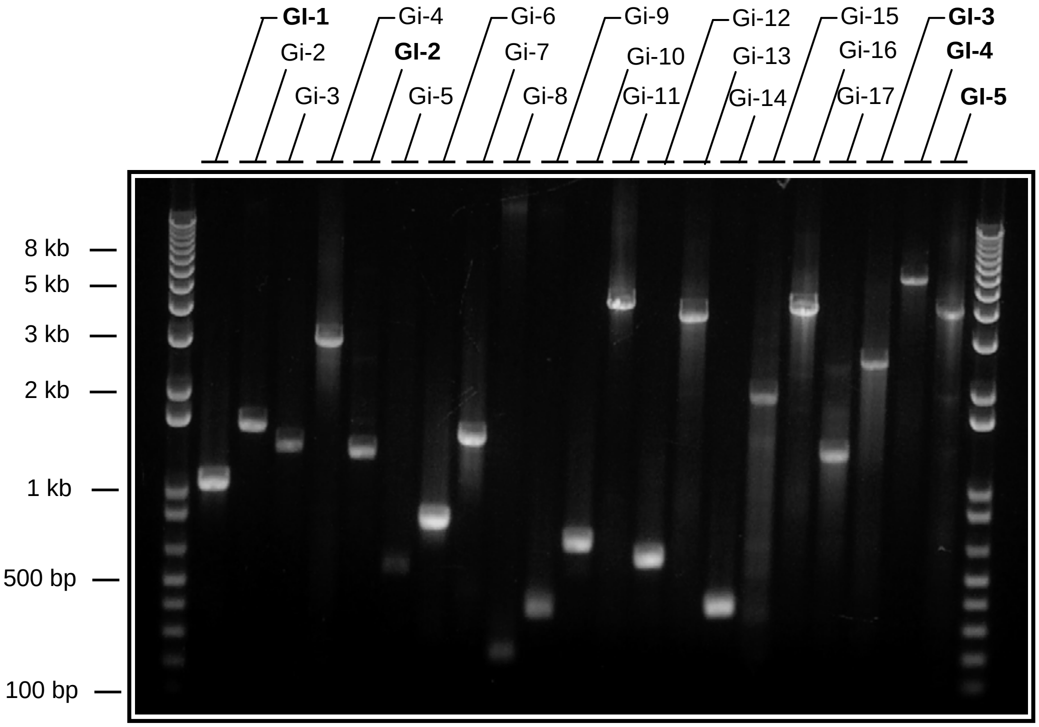

Figure S1: Gel electrophoresis image of PCR products amplified from *S. lividans* genomic DNA using primers listed in Table 1
